# Protecting China’s major urban bird diversity hotspots

**DOI:** 10.1007/s13280-023-01943-z

**Published:** 2023-10-26

**Authors:** Li Li, Mingxiao Yan, Yixuan Hong, Weijia Feng, Dong Xie, Emilio Pagani-Núñez

**Affiliations:** 1 Present Address: Department of Health and Environmental Sciences, Xi’an Jiaotong-Liverpool University, Suzhou, China; 2 Present Address: College of Biology and the Environment, Nanjing Forestry University, Nanjing, China; 3 Present Address: Centre for Conservation and Restoration Science, School of Applied Sciences, Edinburgh Napier University, Edinburgh, UK; 4 Present Address: School of Design, Southern University of Science and Technology, Shenzhen, China; 5 Present Address: School of Ecology, Sun Yat-sen University, Guangzhou, China

**Keywords:** Bird conservation, Convention on biological diversity, COP 15, GBF Target 12, Kunming-Montreal Global Biodiversity Framework, Urban biodiversity

## Abstract

The *Kunming-Montreal Global Biodiversity Framework* puts forward a new conservation target to enhance urban biodiversity. Cities have a great potential for sustaining biodiversity and nurturing a healthy relationship between people and our nearest nature. It is especially important in developing countries such as China, which has a rich biodiversity and a rapidly growing urban population. Using citizen science data, we show that 48% of the national bird diversity and 42% of its threatened species have been recorded in the top-20 most avian-diverse cities of China. Urban bird diversity hotspots clustered along the eastern coast, indicating the importance of establishing an inter-city conservation network along the East Asian-Australasian Flyway. This urban conservation network would be a starting point to promote social recognition of biodiversity’s relational value in a country with a vast population and an increasingly important role in meeting UN’s Sustainable Development Goals.

**Supplementary Information:**

The online version contains supplementary material available at 10.1007/s13280-023-01943-z.

## Introduction

The Conference of the Parties (COP15) of the Convention on Biological Diversity (CBD) has taken place in December 2022 to develop the *Kunming-Montreal Global Biodiversity Framework* (GBF). The GBF negotiation finally agreed upon four goals and 23 targets including the ambitious “30 by 30” agenda, i.e. 30% of terrestrial and marine areas must be protected and managed by the year 2030 (CBD 2022). Achieving the GBF’s ambitious goals requires more efforts to protect human-dominated landscapes with high species richness (Maxwell et al. 2020; Alves-Pinto et al. 2021). Biodiversity should not only be associated with charismatic flora and fauna in pristine habitats, but biodiversity must also be appreciated and protected within human landscapes (Dearborn and Kark 2010). Notably, the GBF adopted a target of urban nature for the first time—Target 12 aims to promote biodiversity-inclusive urban planning through expanding and connecting urban green and blue spaces (CBD 2022). Currently, more than half of the global population lives in urban areas, and the number is projected to reach two thirds by 2050 (Ritchie and Roser 2018). Urban areas can function as “biodiversity arks” (Shaffer 2018), and act as stepping stones to increase habitat connectivity (Saura et al. 2014). There is also evidence that biodiversity can be beneficial for mental health of urban dwellers (Aerts et al. 2018; Methorst et al. 2021). In fact, for most people, the earliest and most frequent experiences with nature take place in urban green spaces. In this regard, protecting urban biodiversity has far-reaching social and cultural implications.

China is one of the world’s 17 megadiverse countries (Mittermeier 1997), with 2340 terrestrial vertebrate species (Liu et al. 2003, 2018). Until 2021, China has protected most of its intact ecosystems: protected areas make up to 18% of China’s land, including the 35 priority areas for biodiversity protection, 28.8% of China’s terrestrial area is covered under the national policy of biodiversity conservation (The State Council Information Office of the People’s Republic of China 2021). Most protected areas concentrate in the less-populated western side of the country rather than in the more urbanized East (Xu et al. 2017). During the same period of increasing protected areas, China has also witnessed the most rapid urbanization of the world (Seto et al. 2012). It has five of the global top-20 most populated cities, namely Shanghai, Beijing, Chongqing, Tianjin, and Guangzhou. By 2020, Beijing had recorded more than 500 species of birds, nearly accounting for a third of China’s total (Townshend 2020). A study on avian diversity in 38 Chinese university campuses recorded 393 bird species, of which 39 of them were endangered, suggesting great potential for urban biodiversity conservation (Zhang et al. 2018). However, homogenous urban environments often lead to increased biotic homogenization of bird communities nationwide (Sun et al. 2022). In their global-scale study, Hughes et al. (2022) showed that effective urban green space management can significantly enhance the capacity of large cities to support high bird diversity. Locke et al. (2019) advocate for placing equal emphasis on lands that encompass urban biodiversity and the diverse values associated with it, even if its contribution to percentage targets will be small. Based on this proposition, we are interested to know: What are the nationwide patterns of urban bird diversity in the country? Where are hotspots of China’s urban bird diversity? Such knowledge is essential to inform the planning of transformative actions required to create synergy between China’s urbanization and biodiversity agendas (O’Meara 2021; Peng et al. 2021).

Unfortunately, urban biodiversity research is a developing field in China (Wu et al. 2014). For a long time, systematic monitoring of urban biodiversity has been lacking, and citizen science data are helping to fill this gap (Callaghan et al. 2020). Citizen science data are increasingly being used to illustrate spatial and temporal patterns of biodiversity for conservation planning at large scales (Schuster et al. 2019; Li et al. 2020). Here, building upon the best available data (Li et al. 2020; Sun et al. 2022), and controlling for socio-economic factors potentially shaping urban biodiversity such as population and city size, GDP, or green urban spaces (Beninde et al. 2015; Chamberlain et al. 2019; Hassell et al. 2021), we identified urban bird diversity hotspots of China. Acknowledging the value of human-dominated areas for conservation, we aimed to understand which urban areas of the country harboured the highest avian diversity, and in which way, this knowledge can be integrated in China’s future conservation plans.

## Materials and methods

### Data sources

We downloaded urban avian diversity data from Bird Report, which is a citizen science database providing georeferenced avian species lists (namely “reports”) collected by volunteers all around China (birdreport.cn). Bird Report data is of high quality because species’ lists are recorded by experienced birders. Moreover, there is a control mechanism consisting in having reviewers check the accuracy of the reported location and species and remove questionable entries. Reviewers are only qualified after submitting more than 300 avian species and 100 reports. To further enhance data quality, we screened all the species in each city and removed those that appeared outside their established distribution limits in the reference handbook of the birds of China (Zheng 2005). From this species pool, we used two additional handbooks to remove species that had less than 20 records and were outside their distribution ranges according to both MacKinnon et al. (2000), and Liu and Chen (2021), resulting in the removal of 125 species’ records across all cities included in our sample.

We screened and selected Chinese cities by population size in 2020 (National Bureau of Statistics of China 2020). According to this criterion, we selected 45 cities having more than 3 million people. While this procedure may introduce biases (see e.g. Kendal et al. 2020), we considered this step important in terms of data quality. Smaller cities rarely have a sufficiently large number of reports to perform meaningful analyses, so that we set a threshold of 3 million or 80 reports for a city to be included in our sample. After adding provincial capitals that were not on the list, we increased the number of cities to 66. We checked the number of species’ lists in each city from the Bird Report website (ebird.org.cn) and excluded cities with less than 80 reports. This step shortened the city list from 66 to 60. Setting a threshold of 80 reports enabled us to include Lhasa, capital of the Tibetan Autonomous Region, a representative large city from Western China. Using these criteria, we guaranteed that the number of observations in each city was sufficient to enable us to compare among cities and to obtain a representative sample all around the country. 

We only included districts corresponding to city centres (i.e. other districts corresponding to peri urban areas were excluded). In China, metropolitan areas are divided into districts: some correspond to the city centre and others represent nearby (smaller) cities and rural areas that are integrated into this large administrative unit. Here, we only used information from city centres for all the variables employed in this study. Only two cities of all those considered in this study represented a problem from the point of view of administrative boundaries: Zhongshan, in Guangdong, and Hong Kong SAR. These cities are not subdivided in districts and the entire metropolitan areas have been merged into a single district. Still, we kept them in our analysis despite their values being overestimated to some extent compared to other cities. Since we controlled by city area and sample size, we guaranteed that comparisons among cities were reasonable and meaningful.

Then, we combined species’ records from all the lists in city centre districts for each city. We downloaded the records of Taipei and Hong Kong SAR from eBird (ebird.org) (Sullivan et al. 2009) because we found a relatively low number of lists in Bird Report. The number of species lists in eBird for these two cities were extremely high (Fig. S1), yet we controlled for this effect when estimating taxonomic diversity. We also downloaded information on city area, GDP, population, and green area from the China City Statistical Yearbook 2020 (National Bureau of Statistics of China 2020). In the case of Taipei and Hong Kong, the same information was obtained from official sources (Taipei City Government 2021; The World Bank 2021). For an overview of this procedure please check Fig. S2.

### Data analysis

We conducted all analyses with R software (R Core Team 2021). We calculated conservation value of the species in each city centre according to IUCN conservation categories (iucnredlist.org), which are internationally recognized estimations of species’ conservation status (Harfoot et al. 2021). We scored each category from 1 to 5 (1: Not Evaluated/Data Deficient/Least Concern, 2: Near Threatened, 3: Vulnerable, 4: Endangered, 5: Critically Endangered/Extinct in the Wild/Extinct). Then, we averaged the scores of all species in each city to represent its conservation value. Averaging the scores per city allowed us to obtain a metric that is conceptually independent of species richness—a city may have many common species and thus have a low score, only cities with a relatively high proportion of threatened species received high scores. Still, the conservation value score had a significant positive correlation with the number of threatened species in each city (*β* ± *SE* = 0.52 ± 0.11, *t* = 4.70, *P* < 0.01) (Fig. S3). 

Citizen science data is subject to biases that need to be assessed and minimized (Kosmala et al. 2016; Callaghan et al. 2020). Here, we detected a markedly uneven number of species’ lists among cities, with cities such as Beijing or Shanghai having thousands of lists while many others having the order of hundreds (Fig. S4A). Moreover, these cities had very different area size (Fig. S4B), which can strongly impact the number of species recorded due to species-area relationships (namely the widespread positive relationship between taxonomic diversity and area) (Callaghan et al. 2021). In this study, we included records of all species detected in a city because we were interested in determining which urban areas of the country may potentially harbour more diversity or can be more important for biodiversity conservation. 

Thus, we standardized the number of avian species in each city by the number of species’ lists and city centre area. To do this, we ran a multiple linear regression using the total number of species in each city as dependent variable and the number of reports and city area (km^2^) as independent variables (Table S1). We took the residuals from this model, namely our standardized proxy of taxonomic diversity, for further analyses. We then assessed sample completeness and data quality using the package iNEXT v2.0.20 (Hsieh et al. 2016). We first randomly selected 80 reports (we sorted all reports by date, divided the total number of reports by 80, and picked up the first report with more than 20 species’ records for each block of reports). We did this using 20 cities spanning the whole range of variation in species richness (we sorted all cities by species richness and picked up one each three cities, i.e. 1st, 4th, 7th, etc.). With these reports, we computed species richness for each city in this subsample. We then assessed sample coverage with ggiNext function and found that for both number of individuals and species richness all these cities showed good sample coverage, reaching 90–100% coverage with a relatively small number of individuals and species (Fig. S5A, B). This suggests that our lower threshold, 80 reports, was sufficient to obtain a representative sample of species richness in a city. Second, we assessed the relationships between observed and estimated (i.e. rarefied) species richness based on these 80 reports obtained with iNEXT function and the total number of species obtained using all the reports in a city. We found that the correlations between these variables were high (observed vs total, *r* = 0.82, *P* < 0.01; estimated vs total, *r* = 0.83, *P* < 0.01). More importantly, we assessed the relationships between observed and estimated species richness in this subsample and the residuals of the model controlling by city area and total number of reports. We found that these residuals were an even more accurate estimation of species richness (observed vs residuals, *r* = 0.92, *P* < 0.01; estimated vs residuals, *r* = 0.92, *P* < 0.01) (Fig. S6A, B). Overall, this suggests that the use of the residuals of a model controlling for the effect of sampling effort and city size on species richness is a reliable and highly representative approach to quantify the diversity of urban avian species based on citizen data. Here, we provide a methodology that can be easily implemented to assess regional and continental patterns of urban biodiversity. This methodology is tailored to obtaining a rapid quantitative assessment tool of broad scale patterns of urban biodiversity.

For our final analyses, we focused on two dependent variables, taxonomic diversity (residuals from the model shown in Table S1) and conservation value (average conservation scores, according to IUCN standards, of all species in each city). In all models, we computed variation inflation factors (VIFs) to assess the risk of false positives due to collinearity and removed any variables with VIF > 2, starting from those variables with the highest score. We used the package car v3.0-11 (Fox et al. 2017) to compute VIF scores. For this reason, we excluded city population size from all models. All the variables were scaled by subtracting the average and dividing by its standard deviation to improve homoscedasticity and model performance. Moreover, we included province as random factor in all models.

We were first interested in ascertaining whether latitudinal and longitudinal patterns of variation existed regarding urban taxonomic diversity and conservation value. To do this, we performed a linear mixed-effect model (LMM) using taxonomic diversity as dependent variable and latitude and longitude as independent variables. LMMs were computed using the package nlme v 3.1-152 (Pinheiro et al. 2007). We repeated this procedure using conservation value as the dependent variable.

We were then interested in determining to what extent cities’ GDP and green space were related to taxonomic diversity and conservation value (Beninde et al. 2015; Chamberlain et al. 2019). To do this, we performed a linear mixed-effect model (LMM) using taxonomic diversity as dependent variable and GDP and green area as independent variables. However, these two independent variables were correlated to each other and had VIF scores higher than 2, so that we constructed these models only using GDP. We repeated this procedure using cities’ bird diversity conservation value as the dependent variable.

## Results

### Urban bird diversity hotspots of China

Taken together, the top-20 most avian-diverse cities have an outstanding record of 714 birds, representing 48% of the total number of avian species recorded in China (1480 native species). Of the 116 threatened species in China according to the IUCN Red List (i.e. Vulnerable status or above), 42% of them (49 species) have been recorded in the top-20 avian-diverse cities.

**Fig. 1:**
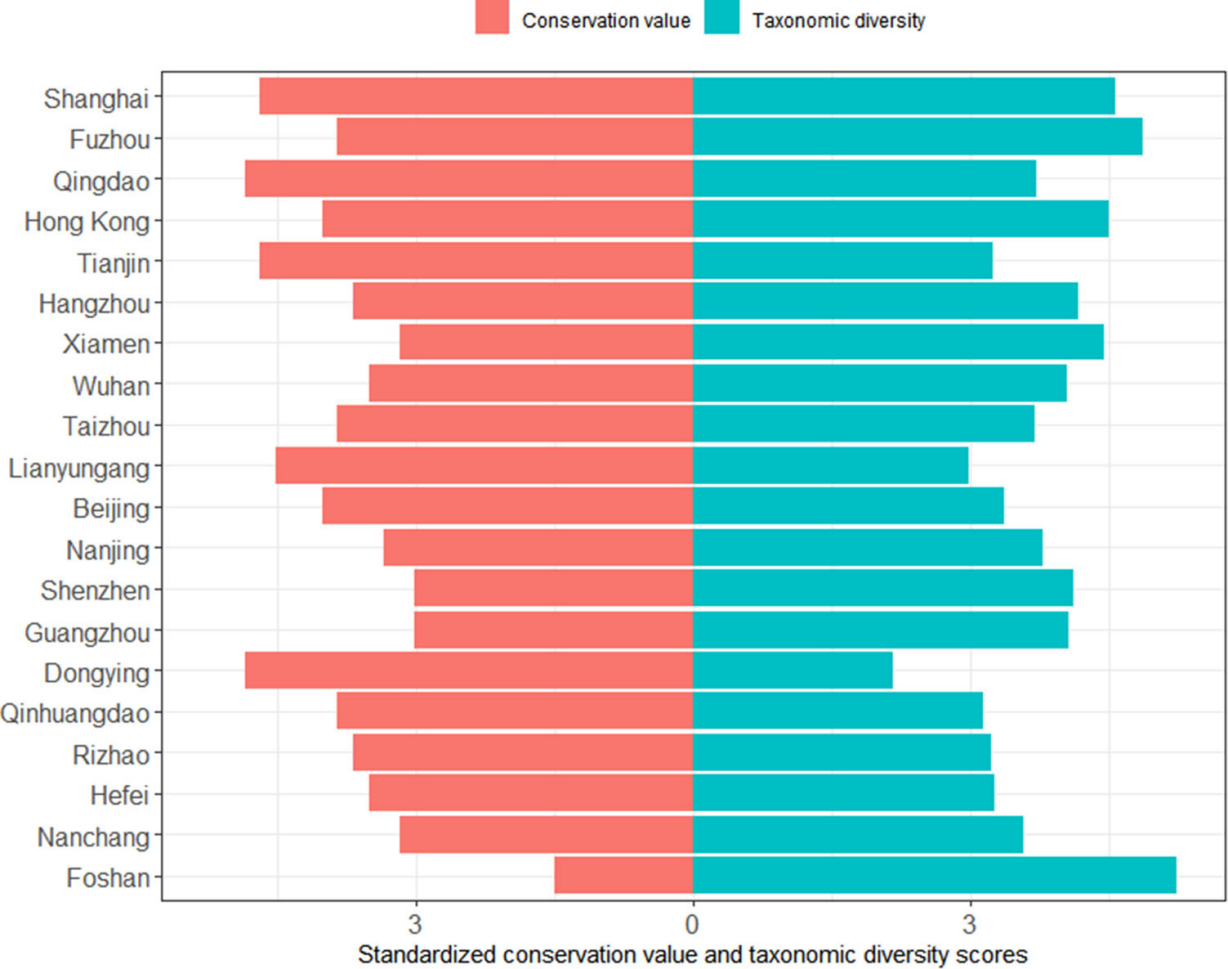
Ranking of the top-20 urban bird diversity hotspots (from top to down) of China. The rank combines both taxonomic diversity and conservation value. Taxonomic diversity scores were computed as the scaled residuals from a regression of number of reports and city area over the recorded number of species per city in Bird Report. Conservation value was estimated by assigning a score to each species according to its IUCN Red List categorization (1 = Lest Concern to 5 = Critically Endangered) and computing the scaled average. Note that both scores were scaled so that they show similar ranges of variation. We summed 1.5 to the scores, as all numbers need to be positive to be properly visualized in the figure

Combining avian taxonomic diversity and conservation value, the top ten urban bird diversity hotspots in China are Shanghai Municipality, Fuzhou (Fujian Province), Qingdao (Shandong Province), Hong Kong SAR, Tianjin Municipality, Hangzhou (Zhejiang Province), Xiamen (Fujian Province), Wuhan (Hubei Province), Taizhou (Zhejiang Province) and Lianyungang (Jiangsu Province) (Fig. 1). The top five most avian-diverse cities are Foshan (Guangdong Province), Fuzhou (Fujian Province), Shanghai Municipality, Hong Kong SAR and Xiamen (Fujian Province). The top five cities with the highest bird conservation value are Qingdao and Dongying (Shandong Province), Shanghai Municipality, Tianjin Municipality and Lianyungang (Jiangsu Province).

### Hotspots in relation with geographical and socio-economic factors

**Fig. 2:**
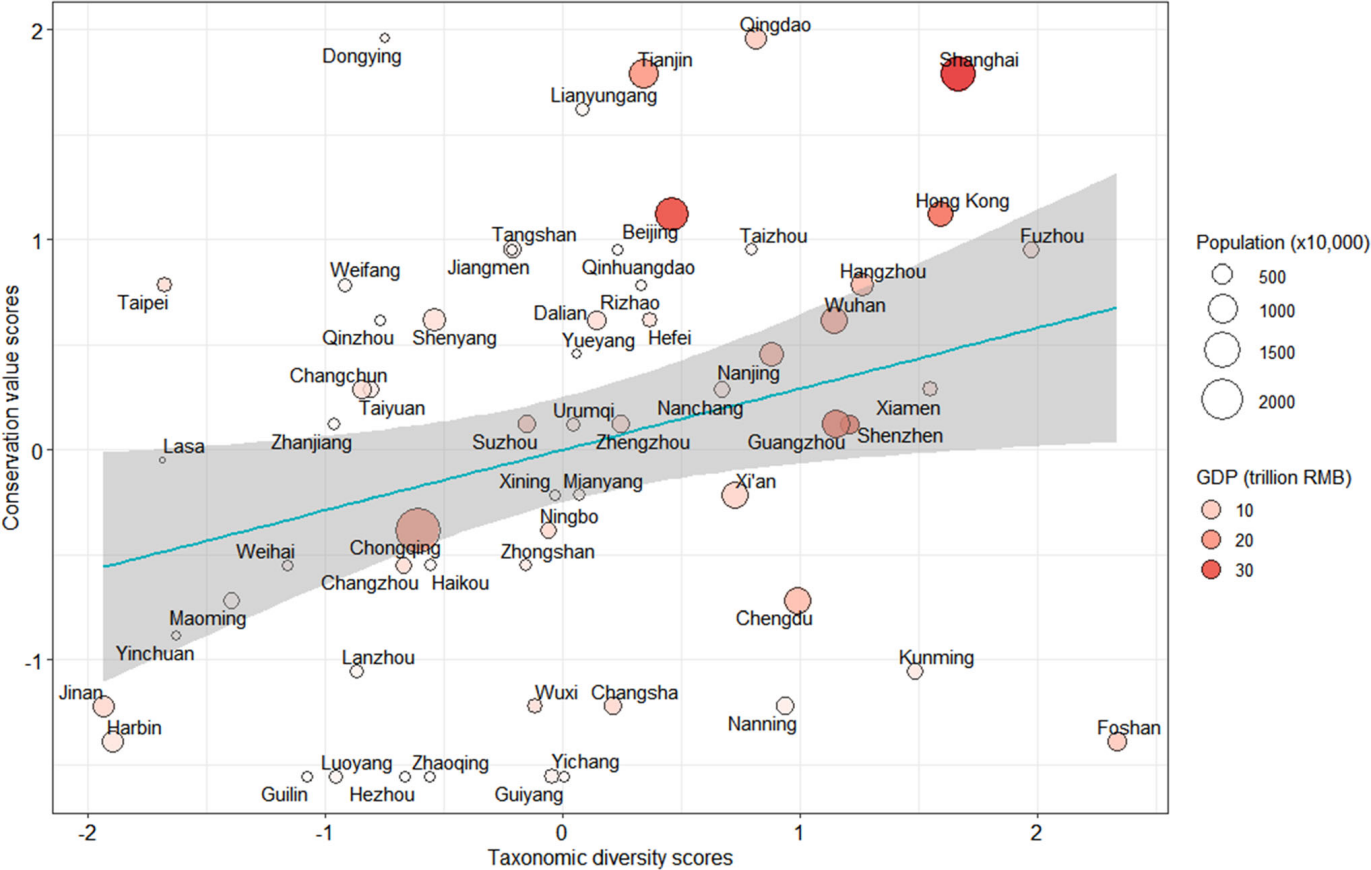
Relationships between bird taxonomic diversity scores and conservation value scores in the top-60 avian-diverse cities of China (*β* ± *SE* = 0.32 ± 0.13, *t* = 2.56, *P* = 0.02) using 137 243 bird species’ lists from Bird Report (ebird.org.cn). Regression line is depicted in blue, and 95% confidence intervals are represented as a grey area. Dot size represents city population size, while the gradient in the hue of red representing city GDP (low GDP lighter red, high GDP darker red)

**Fig. 3:**
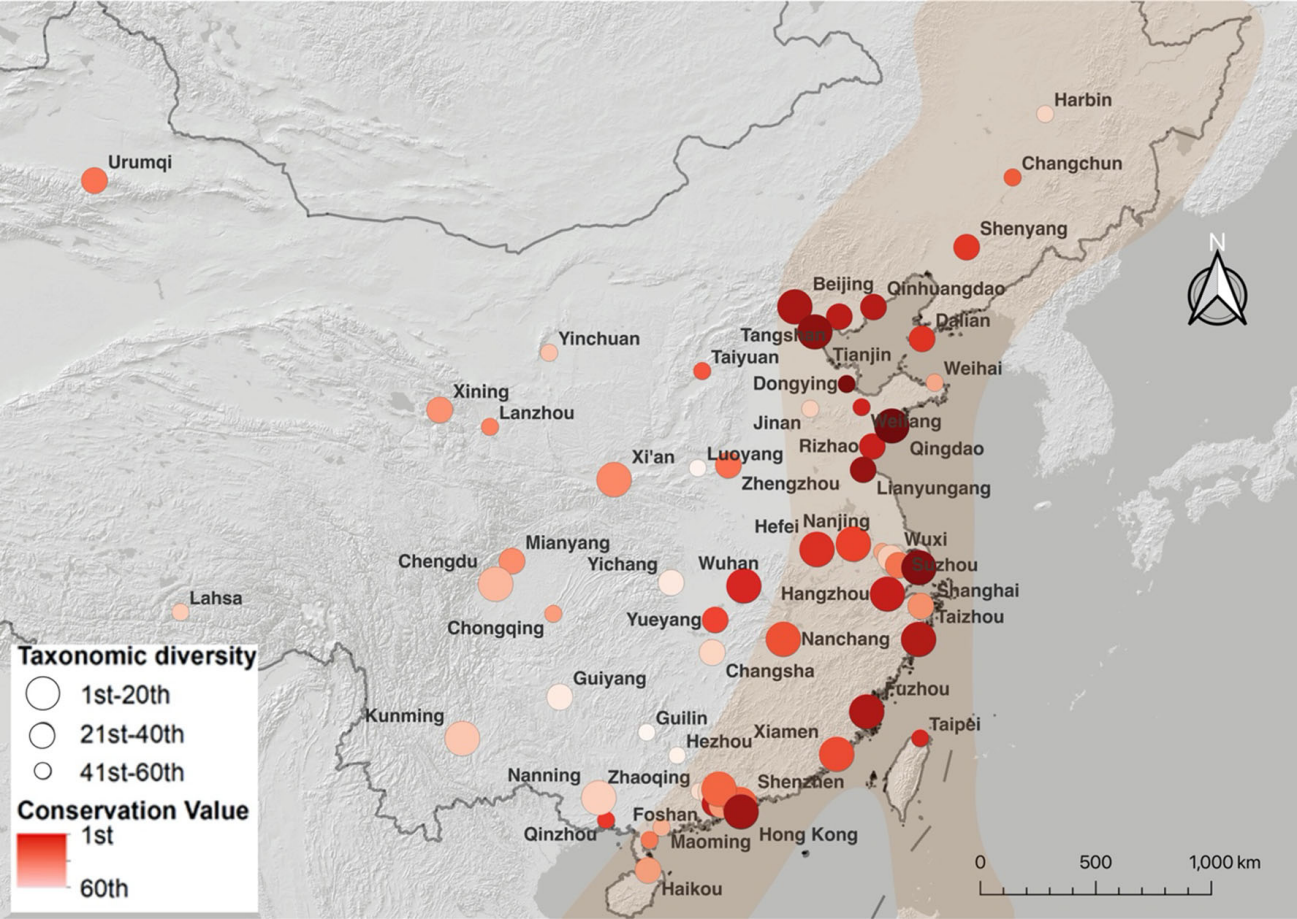
Distribution of China’s urban bird diversity hotspots along the eastern migratory route of China (marked in orange) using 137 243 avian species’ lists from Bird Report (ebird.org.cn). Cities were ranked in function of taxonomic diversity, represented by dot size, and by conservation value, represented in the shades of red (higher conservation value darker red). Taxonomic diversity scores were computed as the scaled residuals from a regression of number of reports and city area over the recorded number of species per city in Bird Report. Conservation value was estimated by assigning a score to each species according to its IUCN Red List categorization (1 = Lest Concern to 5 = Critically Endangered) and computing the scaled average

Taxonomic diversity correlated positively with conservation value across the 60 studied cities (*β* ± *SE* = 0.32 ± 0.13, *t* = 2.56, *P* = 0.02) (Fig. 2). We found pronounced geographical variation in both taxonomic diversity and conservation value of urban birds. Southern cities showed a tendency to be more diverse than northern cities, and eastern cities had higher conservation value than western cities (Tables 1, 2; Figs. 2, 3). Interestingly, both taxonomic diversity and conservation value correlated positively with GDP (Tables 3, 4; Fig. 2). GDP and green space area correlated positively (*β* ± *SE* = 0.88 ± 0.06, *t* = 14.54, *P* < 0.01).

**Table 1:** Results from a linear mixed-effects model using taxonomic diversity as the dependent variable and latitude and longitude as independent variables in 60 cities all around China. Province was set as a random factor. DF = 31

	*β*	*SE*	*t*	*P*
Intercept	< 0.01	0.13	0.02	0.98
Latitude	− 0.30	0.13	− 2.29	0.03
Longitude	0.12	0.13	0.96	0.34

**Table 2:** Results from a linear mixed-effects model using avian diversity conservation value as the dependent variable and latitude and longitude as independent variables in 60 cities all around China. Province was set as a random factor. DF = 31

	*β*	*SE*	*t*	*P*
Intercept	< 0.01	0.12	< 0.01	1.00
Latitude	0.17	0.12	1.38	0.18
Longitude	0.32	0.12	2.55	0.02

**Table 3:** Results from a linear mixed-effects model using taxonomic diversity as the dependent variable and GDP as independent variables in 60 cities all around China. Province was set as a random factor. DF = 32

	*β*	*SE*	*t*	*P*
Intercept	< 0.01	0.11	< 0.01	1.00
GDP	0.49	0.11	4.31	< 0.01

**Table 4:** Results from a linear mixed-effects model using avian diversity conservation value as the dependent variable and GDP as an independent variable in 60 cities all around China. Province was set as a random factor. DF = 32

	*β*	*SE*	*t*	*P*
Intercept	− 0.01	0.14	− 0.10	0.92
GDP	0.33	0.12	2.73	0.01

## Discussion

### Urban bird diversity hotspots cluster along eastern China

The most striking spatial pattern illustrated by our study is that urban bird diversity hotspots cluster along eastern China. Considering the large geographic span of our study, this result diverges from the global pattern where primary productivity is the best indicator for urban bird diversity (Hughes et al. 2022). This is highly related to those cities’ geographic locations—they form a network along the migratory route central to the East Asian-Australasian Flyway. This flyway is the world’s most important migratory route used by almost 400 species (Yong et al. 2021). Among these species, it is noteworthy to mention waders, which could have notably increased the biodiversity scores of cities in this region (Li et al. 2019). For instance, in our data, the top three cities had over 10% of wader species from the total number of species recorded. Migratory birds are among the most threatened taxa (Kirby et al. 2008). Along their migratory routes, artificial light emitted by these large cities likely attracts nocturnally migratory birds and might potentially alter their migratory behaviour (Van Doren et al. 2017). Moreover, future climate change scenarios predict a shift of China’s bird distribution ranges towards eastern China (Hu et al. 2020). Thus, the importance of eastern China for bird biodiversity conservation, a region of the country that remains relatively unprotected (Xu et al. 2017), is likely to become more pronounced even under the most conservative 1.5 °C global warming scenario (Schleussner et al. 2016).

Our results also show that cities with higher GDP and a larger green space area had higher taxonomic diversity and conservation value. This is in sharp contrast with previous studies suggesting negative associations between income and urban biodiversity footprint (Koslowski et al. 2020). The top-20 avian-diverse cities contribute a quarter of China’s GDP and sustain a fifth of China’s urban population. This implies that a substantial share of China’s bird diversity exists in the richest and most populated cities of the country. These cities have a total area of 9678 km^2^ of green space, which corresponds to a 0.1% of China’s terrestrial land. These urban green spaces host a bird diversity that is disproportionally high for the land area they cover. Although we were unable to tease apart the relative effects of GDP and green space area, our results imply that the level of economic development in the most urbanized regions does not seem to compromise their potential for conservation. This result supports the “luxury effect” hypothesis, which suggests that wealthy neighbourhoods may sustain a higher level of biodiversity than those that are less affluent (Chamberlain et al. 2019). The finding has a far-reaching implication for China to achieve the GBF’s Target 12: conservation policies should aim to promote access to and equitable sharing of biodiversity’s benefits among urban residents.

In a global study of 54 cities, Aronson et al. (2014) found these cities collectively sustain approximately 20% of the world’s bird species, and only 14% of the cities in their study host threatened and endangered species. In comparison, our results indicate that those high biodiversity cities of China not only have high bird diversity but also significant conservation value. This requires a focused and integrated conservation effort regardless of how much urban biodiversity contributes to meeting the GBF’s percentage targets. This is particularly important because densely populated urban areas harbouring high biodiversity can be a key source of human-wildlife conflicts. For instance, Peng et al. (2021) suggested that both the Pearl River Delta and Yangtze River Delta have high conflict risk between biodiversity conservation and human land use. Our study further recognizes the importance of the Yellow River Delta region for migratory birds. Taken together, the top-20 urban bird diversity hotspots harbour over one hundred million people and are in the wealthiest provinces of the country. Promoting conservation ideals and biodiversity-friendly behaviours in these cities can maximize transformative changes fostered by governmental and societal conservation efforts. The establishment of these special urban bird conservation areas in China could address the calls for more streamlined protected area management and revisions to the current protected area classification scheme (Xu et al. 2019). This novel protected area type could fall in a new category focusing on safeguarding biodiversity to guaranteeing relevant ecosystem services (Xu et al. 2017).

### Developing a network of cities for bird conservation in China

We propose here the development of a conservation network of China’s top-20 avian-diverse cities. First, this urban bird diversity network should aim to connect isolated habitats within and between cities along the East Asian-Australasian Flyway, both nationally and ideally also internationally. Habitat fragmentation is one of the main threats to biodiversity (Haddad et al. 2015; Liu et al. 2019), so that this network could be a way to enhance connectivity between suitable urban habitat patches for biodiversity. It aligns with the Target 12’s aim to “significantly increase the…connectivity of…green and blue spaces in urban and populated areas” (CBD 2022). Increasing connectivity and improving habitat quality in urban landscapes are particularly crucial for migratory birds due to mis-synchronized resource availability in cities compared to nearby rural areas. For instance, the higher temperatures in urban areas has resulted in early arrival of migrant birds to cities (Tryjanowski et al. 2013). Second, the network could also be designed to increase the connectivity among high-bird-diversity urban areas and surrounding natural and semi-natural spaces, especially high-avian-diversity farmlands and protected areas (Li et al. 2020). Along this migration route, the ecological network containing stepping-stone habitats will support metapopulations of the threated species, sustaining rare but crucial dispersal events (Saura et al. 2014; Millsap 2018; Locke et al. 2019). This would also maximize the efficacy of protecting relatively small and fragmented areas at the city scale. Third, given China’s rapid urbanization, it is essential to thoroughly assess the anthropogenic threats to the 49 threatened species in these highly biodiverse cities. Potential anthropogenic threats should be mitigated with appropriate policies, and these strategies should be incorporated into the process of urban planning (Albert et al. 2020). For example, it would be important to design dark infrastructure (Sordello et al. 2022) and bird-friendly buildings (Yang et al. 2021) to mitigate light pollution and bird collision to glazed building façade during the migrating seasons.

Thanks to the abundant species pool along the migration route, habitat restoration projects that adopt an ecosystem-based approach (CBD 2021) could result in a significant net gain of species both locally and regionally within the network. There is a great potential for ecological restoration in urban areas by increasing suitable habitats for wildlife (Klaus 2013), or by optimizing their spatial configuration. Near the top one urban bird diversity hotspot of Shanghai, Tianfu National Wetland Park has recorded over 200 avian species in a 50-hectare restored habitat. In 2016, Tianfu was still an abandoned horse ranch surrounded by rice paddies. Suzhou municipal government designed a restoration project to create connected ponds with a range of water levels. Within five years, 60% more birds (77 species, particularly a high functional diversity of waterbirds) have been observed in the park (CSZTV 2021). It is evident how targeted conservation policies can enhance urban biodiversity, potentially benefiting cities along the East Asian-Australasian Flyway.

The benefits of this urban conservation network may also promote healthy urban living. The world has just experienced the largest avian influenza outbreak ever recorded. While live poultry trade, intrinsically linked to rural environments, is usually regarded as the main source of the disease in cities (Verhagen et al. 2012; Ineson et al. 2022), it is interesting to note that protecting wetland ecosystems, an important avian habitat in Chinese cities, has been found to reduce the spread of this disease in China (Wu et al. 2020). Besides, the positive impacts of avian biodiversity on mental health in urban areas, mediated through various mechanisms, such as stress reduction and attention restoration, are well-documented (Methorst et al. 2021). Participation on garden bird counts and generally daily encounters with birds result in long-term improvements in mental health, particularly for people affected by anxiety and depression (Hammoud et al. 2022; White et al. 2023). Neighbourhoods with high vegetation cover and bird abundance have lower prevalence of mental health issues (Cox et al. 2017). The network has the potential to foster synergy among multiple urban sustainable development goals of China, promoting the connection between nature conservation and human well-being.

### Limitations of the study

We must acknowledge limitations of this study. First, what we have labelled as conservation value is often represented as the proportion of species that fall under any of IUCN categories signalling that a species is threatened. However, proportions are problematic from a statistical point of view, and we preferred to use a continuous metric to characterize this feature. Moreover, the IUCN red list is just an estimation of current extinction risk reliant on data quality and availability so we may have underestimated species’ and cities’ conservation value scores. Second, we identified urban biodiversity hotspot at the city scale, but urban biodiversity hotspots might be even localized areas within a city. Further research is needed to determine which exact areas within these highly bird diverse cities merit attention from a conservation point of view. Moreover, it is fundamental to connect urban biodiversity hotspots with biodiversity hotspots outside urban areas, which should receive similar or even more attention. Third, we did not address any potential social and cultural drivers that might have led to high urban bird diversity in eastern Chinese cities (Rega-Brodsky et al. 2022). Finally, avian diversity composition and abundance may vary strongly across seasons. More detailed studies on different taxa and dimensions of biodiversity are required to provide a full picture of the potential conservation value of multi-faceted urban biodiversity in China.

## Conclusions

Protecting urban bird diversity in China has profound social and cultural implications, facilitating the transformative changes needed to meet the *Kunming-Montreal Global Biodiversity Framework’s* targets. The top-20 urban bird diversity hotspots have 22% of China’s urban population, which represent potential conflicts between humans and wildlife but also opportunities to have a positive impact on both people and biodiversity. The launch of the GBF is a timely opportunity for China to pursue a biodiversity-friendly urbanization model, and to create synergies among the 2030 biodiversity targets with other important sustainable development goals. The spatial pattern that high-avian-diversity cities cluster in eastern China also requires a substantial reconfiguration of the current protected area system, in line with novel approaches that have already been suggested elsewhere (Xu et al 2019). Therefore, biodiversity-inclusive urban planning could promote positive encounters of urban dwellers with wild species (Palliwoda et al. 2017), reinforcing relational values derived from interactions with nature (Schröter et al. 2020). It may forge a deep understanding in the society that biodiversity also exists in our urban centres, providing irreplaceable ecosystem services to citizens, and contributing positively to people’s mental and physical health (WHO 2015; Methorst et al. 2021). This change in how people perceive human–nature relationships will eventually pave the way for a bottom-up change regarding how biodiversity is culturally constructed in China and around the world. This normative change is not a trivial matter. Essentially, the success of this new conservation agenda depends on the attitude and action of the majority of the world’s population.

### Supplementary Information

Below is the link to the electronic supplementary material.

Supplementary file1 (PDF 406 KB)

## Data Availability

The datasets generated during the current study *are available from the corresponding author on reasonable request*.
