# Supplementary material for: Protecting China’s major urban bird diversity hotspots
Source: Ambio. 2023 Oct 26;53(2):339–50. doi: 10.1007/s13280-023-01943-z (PMC10774474; doi:10.1007/s13280-023-01943-z)
Supplement: Supplementary file 1 — Supplementary file1 (PDF 406 KB) [file 13280_2023_1943_MOESM1_ESM.pdf]

Ambio

Electronic Supplementary Material

This supplementary material has not been peer reviewed.

Title: **Protecting China's major urban bird diversity hotspots**

Authors: Li Li, Mingxiao Yan, Yixuan Hong. Weijia Feng, Dong Xie, Emilio Pagani-Núñez

Index

Figure S1 – Species lists from each city
Figure S2 – Flowchart diagram of our methodology
Figure S3– Relationships between conservation value score and the number of threatened species in each city
Figures S4A&B – Species lists and city area histograms
Figure S5A&B – Species accumulations curves
Figure S6A&B – Relationships between iNEXT rarefied diversity estimates and species richness standardized by number of reports and city area
Table S1 – Model to standardize species richness by the number of species lists and city area

Figure S1. Number of avian species' lists gathered from the Bird Report website (bridreport.org) in 60 cities of China. Cities are sorted by the number of lists. The left panel zooms in the cities with fewer reports. The right panel shows the full list.

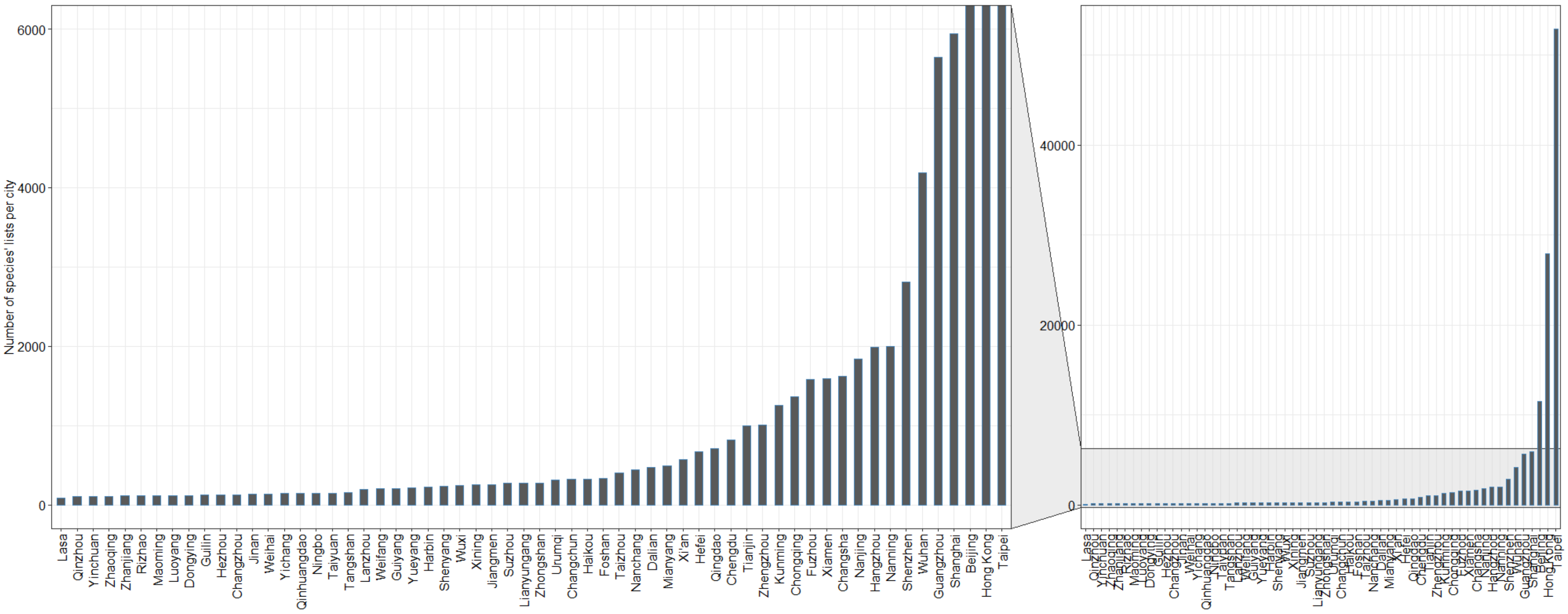

Figure S2. Flowchart diagram illustrating the methodology followed in this study.

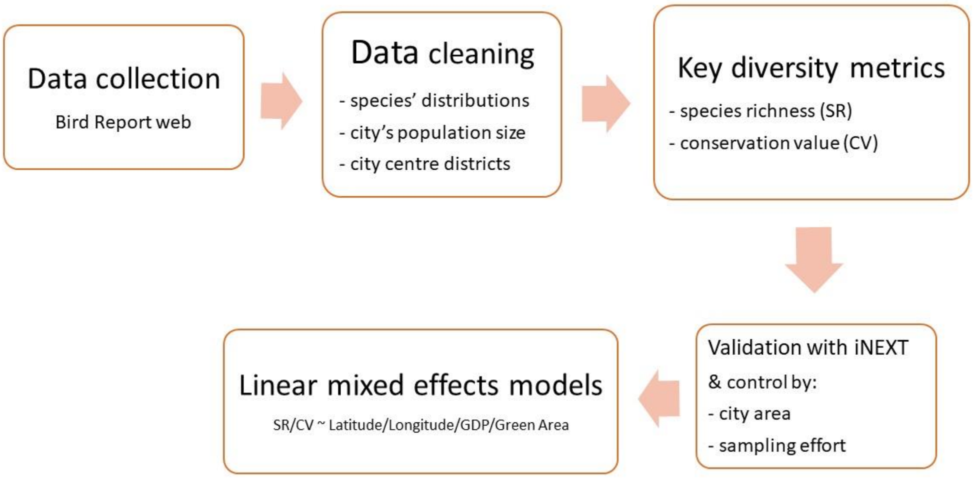

Figure S3. Relationships between conservation value score and the number of threatened species in n the top-60 biodiverse cities of China recorded in Bird Report (birdreport.org). Regression line is provided, as well as 95% confidence intervals represented as a grey area. Both variables were scaled.

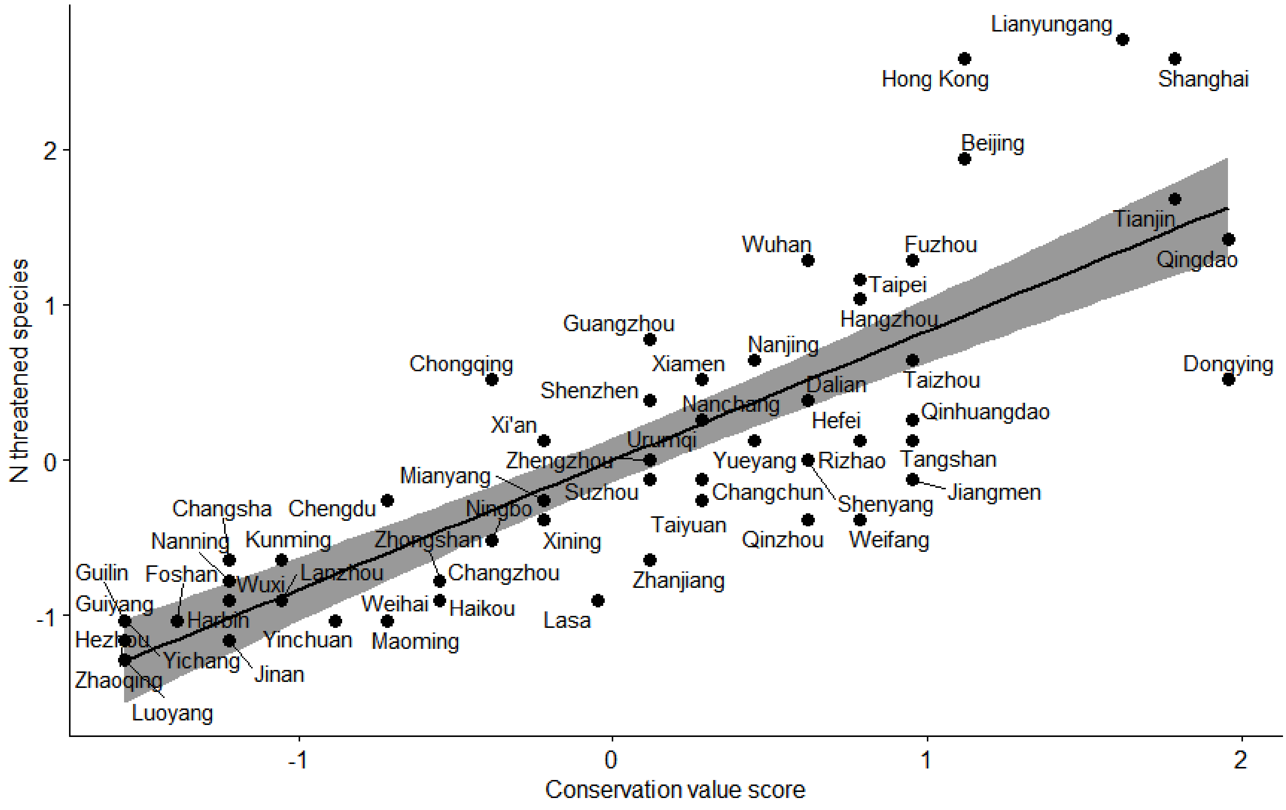

Figure S4. **A)** Data distribution of cities by the number of species' lists. Most cities had around 100 lists; the number of cities with lists in the thousands of species was considerably smaller. The X-axis has been log transformed. **B)** Data distribution of cities by city area (km^2). The city centre with the largest area was Chongqing municipality (43263 km^2), followed by Beijing municipality (16406 km^2). The city with the smallest area was Taipei (272 km^2). Note that Chongqing had a disproportionally large city centre area probably due to its special nature as municipality directly managed by the central government.

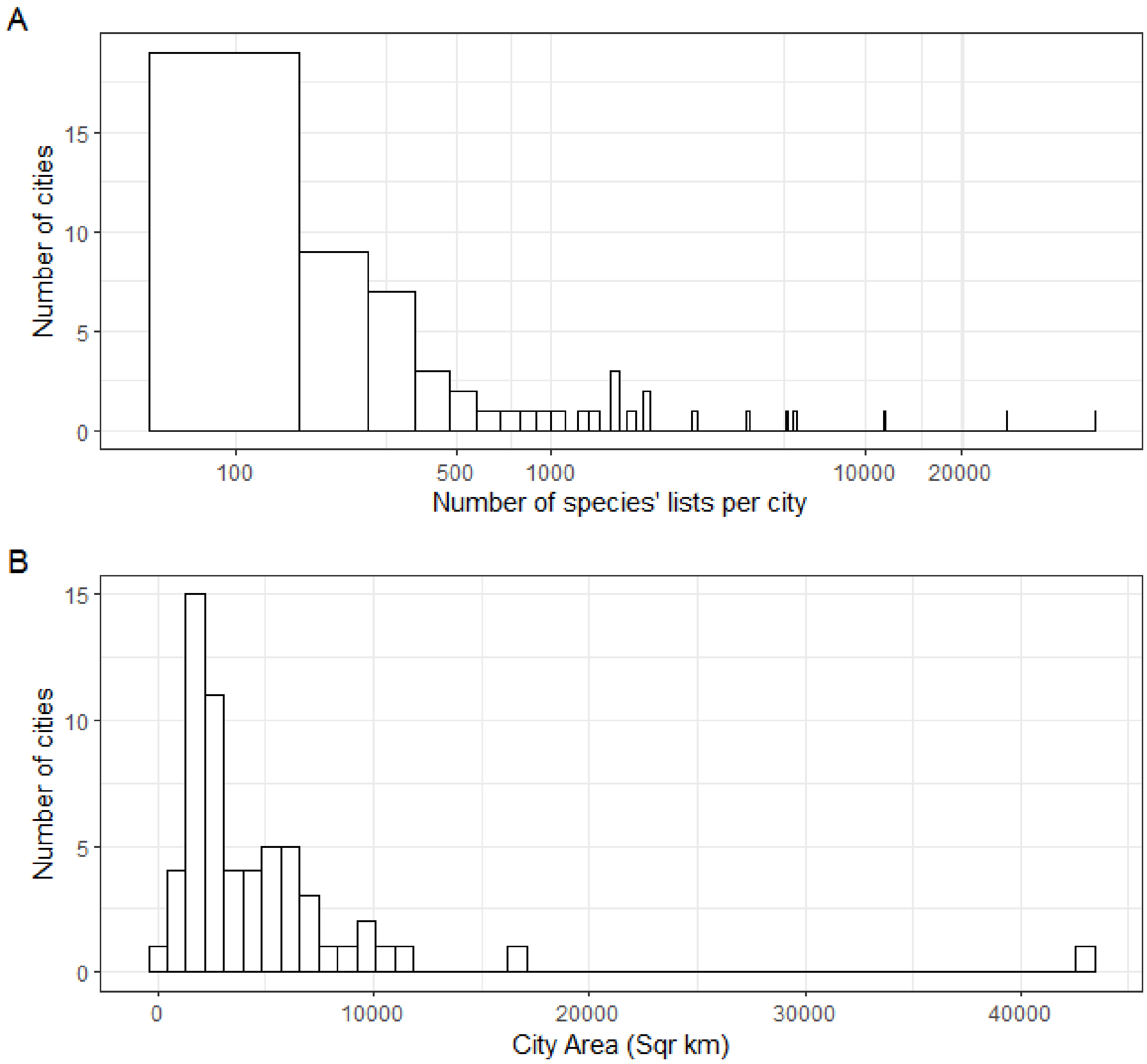

Figure S5. A) Species accumulation curve based on number of individuals recorded & **B)** Sample coverage (N species detected) based on sampling effort. Solid lines are based on the recorded data and dashed lines based on the extrapolation made by iNEXT algorithm. Shaded areas represent 95% confidence intervals. Plots based on a subsample of 20 cities covering the whole range of species richness for which we randomly selected 80 reports (bird lists).

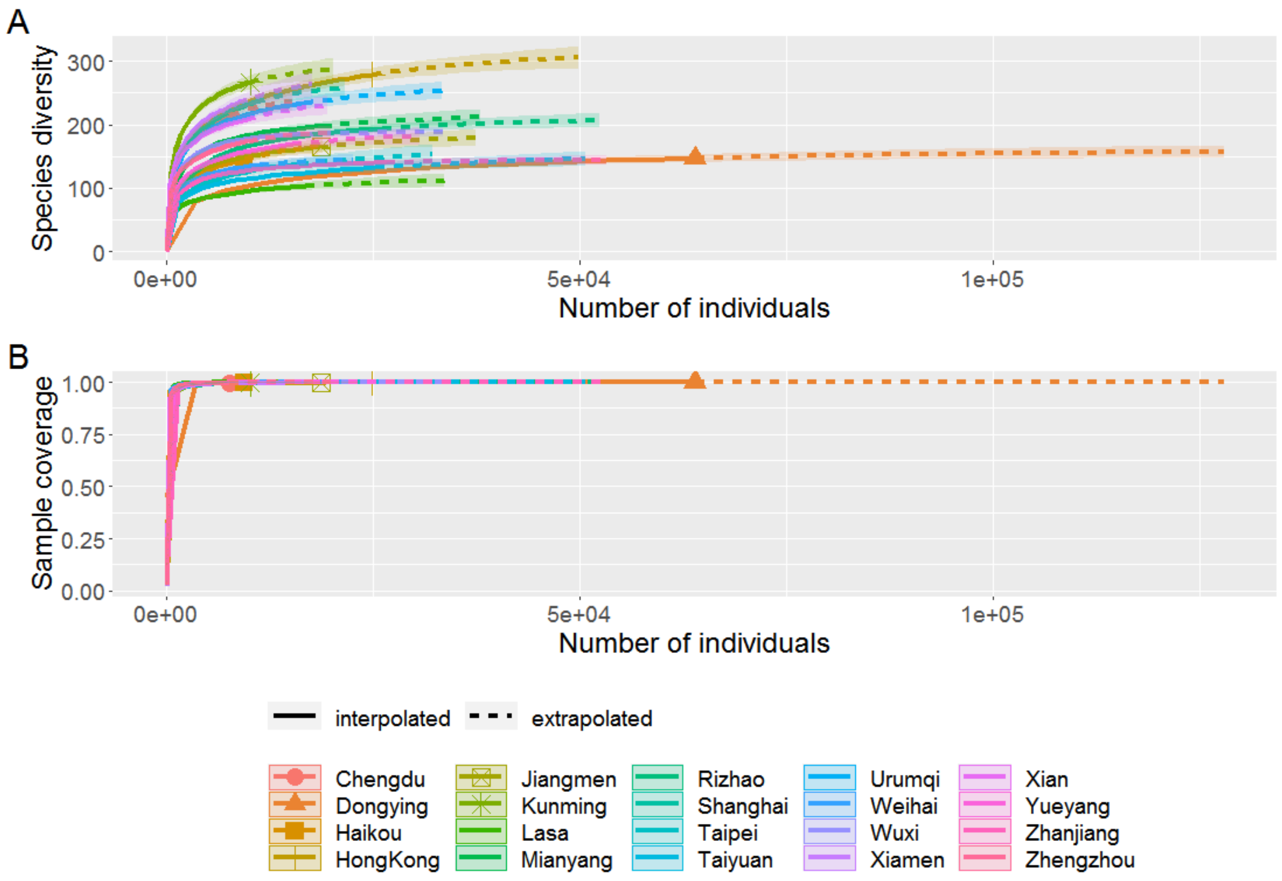

Figure 6. A) Relationships between observed species richness in a subsample of 20 cities based on 80 randomly selected bird reports and residuals from a model using species richness based on all the records per city as dependent variable and number of reports and city area as independent variables. **B)** Relationships between rarefied richness computed using iNEXT package and based on a subsample of 80 randomly selected reports from 20 cities and residuals from the model described above. All variables were scaled.

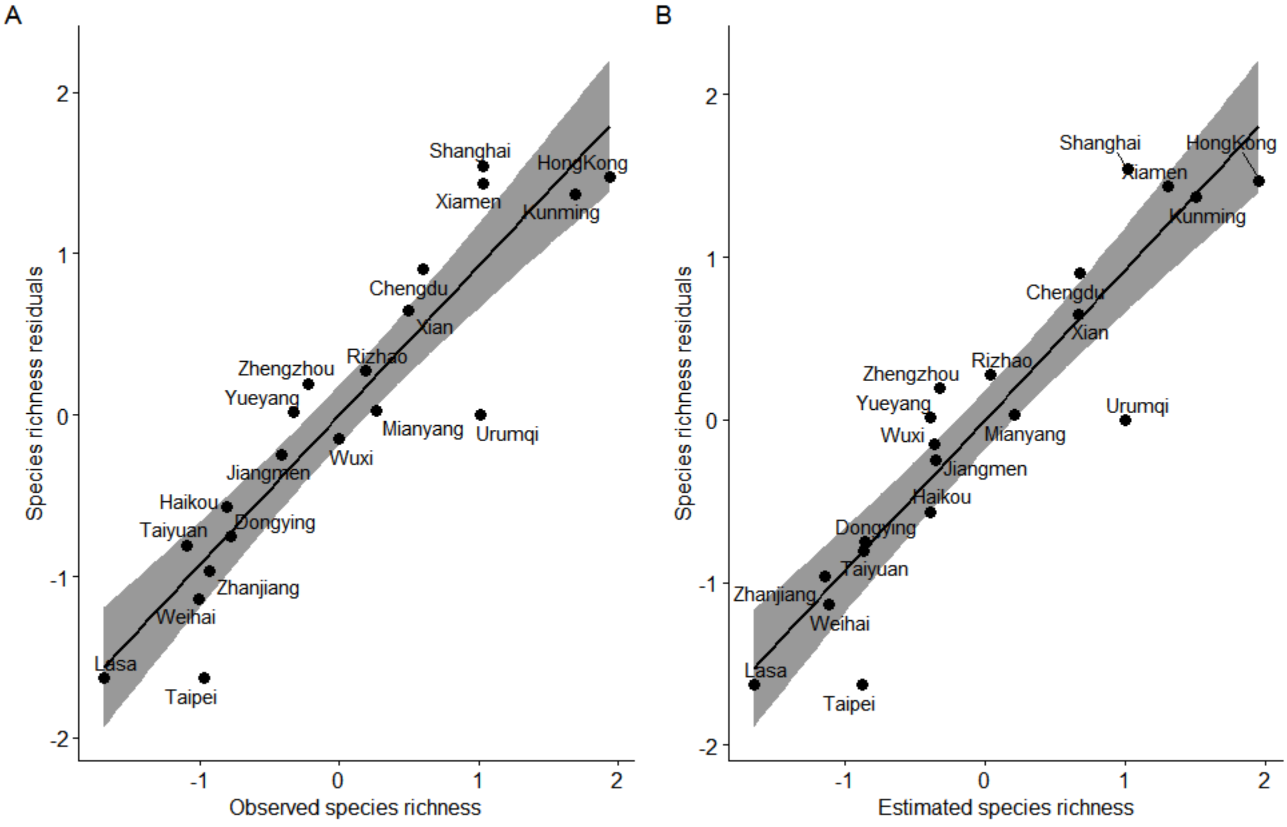

Table S1. Results from a multiple linear regression using the total number of species across 60 Chinese cities recorded in Bird Report (birdreport.org) as dependent variables and the number of species' lists and city area (in km^2, namely the area of the urban districts in each city) as independent variables. Adjusted R^2 = 0.34. The three variables were scaled.

	β	*SE*	*t*	*P*
Intercept	224.10	12.94	17.31	<0.01
N species' lists	0.47	0.11	4.32	<0.01
City area	0.34	0.11	3.16	<0.01
